# Use of progression criteria to support monitoring and commissioning decision making of public health services: lessons from Better Start Bradford

**DOI:** 10.1186/s12889-019-7149-7

**Published:** 2019-06-27

**Authors:** M. Bryant, N. Dharni, J. Dickerson, K. Willan, R. McEachan, J. Duffy, M. Howell

**Affiliations:** 1 Clinical Trials Research Unit, University of Leeds, Leeds, LS29JT UK; 2 Bradford Institute of Health Research, Bradford Teaching Hospitals NHS Foundation Trust, Bradford, BD9 6RJ UK; 3 Bradford Trident Charity and Social Enterprise, Park Lane, Bradford, BD5 0LN UK

**Keywords:** Implementation, Monitoring, Early years intervention, Child health, Obesity, Language, Social, Emotional

## Abstract

**Background:**

Commissioning and monitoring of community-based interventions is a challenge due to the complex nature of the environment and the lack of any explicit cut-offs to guide decision making. At what point, for example, is participant enrolment to interventions, course completion or satisfaction deemed to be acceptable or sufficient for continued funding? We aimed to identify and quantify key progression criteria for fourteen early years interventions by (1) agreeing the top three criteria for monitoring of successful implementation and progress; and (2) agreeing boundaries to categorise interventions as ‘meeting anticipated target’ (green); ‘falling short of targets’ (amber) and ‘targets not being met’ (red).

**Methods:**

We ran three workshops in partnership with the UK’s Big Lottery Fund commissioned programme ‘Better Start Bradford’ (implementing more than 20 interventions to improve the health, wellbeing and development of children aged 0–3) to support decision making by agreeing progression criteria for the interventions being delivered. Workshops included 72 participants, representing a range of professional groups including intervention delivery teams, commissioners, intervention-monitoring teams, academics and community representatives. After discussion and activities, final decisions were submitted using electronic voting devices. All participants were invited to reconsider their responses via a post-workshop questionnaire.

**Results:**

Three key progression criteria were assigned to each of the 14 interventions. Overall, criteria that participants most commonly voted for were recruitment, implementation and reach, but these differed according to each intervention. Cut-off values used to indicate when an intervention moved to ‘red’ varied by criteria; the lowest being for recruitment, where participants agreed that meeting less than 65% of the targeted recruitment would be deemed as ‘red’ (falling short of target).

**Conclusions:**

Our methodology for monitoring the progression of interventions has resulted in a clear pathway which will support commissioners and intervention teams in local decision making within the Better Start Bradford programme and beyond. This work can support others wishing to implement a formal system for monitoring the progression of public health interventions.

## Background

Early years’ interventions focus on optimising outcomes for children through the provision of services from conception to age 7 years. In the UK, ‘commissioning’ describes the process of assessing needs, planning and prioritising, purchasing and monitoring health services. The commissioning for early years services has to consider multiple elements, including understanding local needs and existing provision, identifying and understanding what the key outcomes are, design and procurement of services and monitoring their performance and impact [1, 2]. This is often a challenge, due to the complex nature of the environment and political landscapes. Decisions to commission or de-commission interventions should be based on the best available evidence; however, for many, evidence is lacking or not appropriate to local contexts. Rather than adopting standard processes to inform decisions, judgements are driven by a variety of factors that are often highly political, personal and relational. These are often influenced by hierarchical power and can lead to tensions between stakeholders, [3, 4], with decision making becoming a social process; of which costs are a key factor [5].

It has been argued that the lack of randomised controlled evidence does not necessarily mean that robust decision-making methods cannot be applied [6] and that methods such as theory-based approaches, mechanistic evidence, observational data and causal models should be considered. Such approaches utilise data to hypothesise the likelihood that an intervention will lead to improvements in outcomes. In practice, given the lack of appropriate data, the timescales for commissioning and the required resources, decision-making is often based on monitoring and summative data. These can be used instead of, concurrently with, or in preparation for assessing outcomes [7]. Well conducted monitoring and summative evaluation is also important for supporting necessary improvements to interventions and / or their implementation. Without ongoing monitoring of services, a ‘test and learn’ approach is not possible, and necessary changes to optimise services cannot be delivered. As a result, many interventions may well have been categorised as failing even when there was potential for them to succeed.

Frameworks exist to support the collection of data for monitoring and summative evaluation (e.g. [8–10]) which include measurement of process type data such as recruitment, reach and fidelity. In reality, the complexity of most early years interventions results in far more data than can be regularly reviewed by commissioning bodies; or worst still, no data. The first challenge, therefore, is to decide which components are most important, and evaluate fewer components well, rather than many poorly. Selection of components, or ‘key criteria’ should be based on intervention objectives and logic models where available (describing how each intervention component has potential to impact on outcomes), with decision-making shared between all stakeholders to facilitate greater ‘buy-in’. However, once selected, it is difficult to define what ‘success’ actually looks like and whether or not monitoring data suggest that an intervention can progress, needs more support, or should be de-commissioned.

Similar consideration for progression is applied in clinical trials research to define whether or not early phase trials (pilot and feasibility trials) indicate that larger definitive trials should be conducted [11]. In this field, similar components are considered to those monitored in community based interventions, particularly recruitment and completion rates. Recent guidance supports which components are important in the progression of pilot and feasibility studies to definitive trials [11]. However, there is no similar guidance for decision making for interventions delivered to support the early years (often delivered in community venues) and little agreement regarding what actually constitutes effective implementation. At what point, for example, does public engagement, recruitment and completion become acceptable or sufficient? If a group-based parenting programme designed for ten people regularly has seven attending, is this sufficient? If fidelity is considered adequate 50% of the time, should this be questioned?

### Study aims and objectives

We aimed to identify and quantify key progression criteria for early years interventions through workshops conducted by a multi-stakeholder group within a Better Start Bradford (BSB) [12]. BSB is a 10 year programme funded by the Big Lottery Fund which is implementing more than 20 interventions to improve outcomes for children aged 0–3 years in the three key areas: social and emotional development; communication and language development; and nutrition. The programme is running from 2015 to 2025 within three inner city areas of Bradford. BSB has adopted a test and learn ethos in which each intervention is closely monitored so that they can be re-commissioned, modified or de-commissioned on a 3 year cycle. Interventions were launched over a period of 3 years from 2015 to 2018.

## Methods

We applied the methodology recommended by the Medical Research Council to determine progression criteria to pilot and feasibility studies to support the monitoring of interventions being implemented in early years services [11]. This provided a clear framework to logically consider progression criteria which was deemed to be highly relevant to monitoring interventions, including: Use of Green (go), amber (amend) and red (stop) rather than a stop/go approach; achieving a balance between being firm enough to promote ambition, yet flexible enough to remedy problems; and basing criteria on rates per centre per unit time, rather than an absolute number.

### Workshops

Using the principles of co-production [13], we hosted three one-day workshops in a community location aimed at (1) identifying key progression criteria components for 14 early years interventions being implemented through the Better Start Bradford programme (see Table 1) and (2) agreeing cut-offs to monitor whether interventions would be deemed as ‘Green’ (meeting criteria targets), ‘Amber’ (falling short of targets) and ‘Red’ (targets not being met, instigating actions to resolve issues and decommissioning discussions). Participants representing a range of stakeholders including intervention delivery teams, commissioners, intervention monitoring teams (Better Start Bradford facilitators), academics (topic expert collaborators) and community representatives (via existing community representative groups) were recruited to take part in workshops via direct invitation. Other than membership to one of these stakeholder groups, no further eligibility criteria were applied. As this was deemed to be part of the service auditing and monitoring process, consent was not recorded.

**Table 1:** Early years interventions

HENRY	
A universal group programme to improve healthy eating and physical activity in young children 0-to-4 year olds. Eight weekly sessions are delivered in community settings.	
Perinatal Support Service	
Targeted support for pregnant women and mothers of babies under 1 year old at risk of mild/moderate mental health issues. Includes one-to-one support and signposting to appropriate services.	
Talking Together	
Universal screening for language delay of 2 year olds; an in home programme for parents with children at risk of delay. Aims to foster positive parent-child interactions and supportive home environments that enrich children’s early language development.	
Welcome to the World	
A universal nine-week antenatal course intended to support parents-to-be in the transition to parenthood. Intended outcomes are improved parental wellbeing with less anxiety/depression; confidence in infant care; improved sense of attachment; improved couple relationship; and greater intention to breastfeed.	
Personalised Midwifery Care Pilot	
A statutory offer of personalised midwife care by a named midwife or back up buddy who provides continuity of care for women throughout the antenatal and postnatal periods aimed at improving maternal mental health and optimising satisfaction with the pregnancy, birth and postnatal experience.	
Home-Start Better Start	
Targeted peer support for vulnerable women. A volunteer-based support programme consisting of weekly home visits for venerable families with young children or pregnant women, to provide a ‘helping hand’ to a broad range of factors (including domestic support, friendship and support in a number of situations such as teenage pregnancy, language difficulties, psychopathology, psychosocial problems, substance use, domestic violence, deprivation, isolation). Aims to promote a healthy and supportive family environment for children by improving parents’ coping skills, to ensure healthy child development.	
Better Start Imagine	
Universal book gifting and book sharing sessions. Better Start Imagine provides monthly book gifting to the homes of all 0–4 year olds. It is aimed at improving child-parent interactions, parents’ confidence with books, children’s social interactions and language and communication skills in children.	
Bradford Doulas	
Targeted support in late pregnancy, birth and post-natally for vulnerable women provided by volunteers. Aimed at improving outcomes at each of these stages, including satisfaction, intention to breast feed and attachment.	
ESOL+ for pregnancy	
A targeted English language course for women with little or no English during pregnancy. Aims to help pregnant women whose first language is not English communicate with midwives and doctors, engage with key health messages and learn about British systems and practices.	
Family Nurse Partnership	
Intensive home visiting for vulnerable women aged < 25. Better Start Bradford is one of 11 sites nationally that implements an adapted model of Family Nurse Partnership (FNP): FNP ADAPT (increasing flexibility in eligibility and approach).	
HAPPY	
A targeted, perinatal healthy eating and parenting course for overweight mums with a BMI over 25 kg/m2. Aimed at reducing the risk of obesity in children. It is a 12 session antenatal and post-natal group-based programme delivered in community settings.	
Baby Steps	
A targeted perinatal parent education programme for vulnerable parents. Delivered during and after pregnancy via home visits and eight group sessions.	
Cooking for a Better Start	
A universal cook and eat sessions over 6 weekly sessions aimed at reducing barriers to cooking experienced by some families by providing knowledge, skills, and equipment	
Forest School Play Project	
Universal outdoor play in the natural environment for 2-3 year old children and parents to increase levels of physical activity and reduce the risk of obesity.	

All three workshops were held in a community centre, located in the heart of the Better Start area. Workshop one (November 2016) was focused on determining progression criteria for four interventions (HENRY, Perinatal Support Service, Talking Together, Welcome to the World and Personalised Midwifery Care Pilot) and included a session to consider progression criteria cut-offs which could be applied for all criteria (and hence be used for any intervention). Workshops two (March 2017) and three (February 2018) considered criteria for five (Better Start imagine, Bradford Doula, ESOL+, Family Nurse Partnership and Home-Start Better Start) and four interventions (HAPPY, Baby steps, Cooking for a Better Start and Forest School Play Project) respectively. Ideally, workshops to agree progression criteria would be best placed prior to implementation (i.e. during service design); however many of our intervention were already being implemented prior to workshops.

Workshops lasted 3-4 h. Although some participants attended all three workshops (i.e. academics and Better Start facilitators), intervention delivery team participants, members of the public and local authority representatives differed for each meeting (with invitations sent according to the interventions under discussion); though there was a similar distribution of stakeholders throughout the three workshops. Workshops were attended by 72 participants, (29 in workshop one, 20 in workshop two, 23 in workshop three), including representatives from intervention delivery teams (*n* = 22), commissioners (*n* = 8), intervention monitoring teams (*n* = 19), academics (*n* = 20), and community representatives (*n* = 3).

#### Determining key progression criteria

Participants were first presented with a summary of each intervention, including the objectives, referral pathways, nature of delivery, logic model and previously considered key performance indicators. Through discussion and workshop activities, participants were then asked to consider which progression criteria they considered key to the performance of each intervention. To do this, they were provided with a list of 7 potential criteria, which were derived from progression criteria previously identified by the MRC for trial progression (Table 2). Following discussion amongst groups of 5–6 people, each participant was asked to independently rank the importance of the progression criteria for each intervention, initially through a paper-based exercise and then via an electronic voting system (used to gather additional data on the type of stakeholder and allow instant, anonymous feedback of the outcome for further discussion). Each response was linked to a participant ID, which included information about the type of stakeholder, but no further participant characteristics were recorded.

**Table 2:** Potential progression criteria used to monitor interventions

Progression criteria	Description	Example targets
Recruitment	Family/parent/child enrolment	Number of women enrolled on to a programme per year
Reach	Enrolment of population with intended characteristics	Sample representative of population ethnicity (White British: 25%, Pakistani: 50%, Eastern European: 10%)
Implementation	Activities designed to deliver the intervention	Number of volunteers trained to deliver peer support
Satisfaction	Family/parent/child satisfaction with intervention content and/or delivery	Percentage of parents recommending a course in response to Friends and Family test
Completion	Intervention completion / attendance rate	Proportion of families attending at least 5/8 sessions
Fidelity	Extent to which intervention is delivered as intended	Percentage of women receiving continuity of care from a midwife
Data quality	Quality of data routinely collected by intervention teams	Proportion of missing/incomplete data from questionnaires

#### Determining cut-offs to categorise performance of progression criteria

**Fig. 1:**
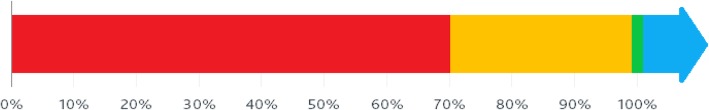
Example vote for cut-offs with 70% chosen as cut-off to indicate targets not being met (i.e. the point at which an intervention goes into red)

After the ranking exercise, participants were asked to agree boundaries to categorise interventions as ‘meeting anticipated target’ (green); ‘falling short of targets’ (amber) and ‘targets not being met’ (red) for all progression criteria. We considered meeting 100% of a target as reaching ‘Green’ status. Rather than using absolute figures (e.g. recruiting 10 families per programme) we applied proportions to permit generalisability across interventions (100% of target recruitment). For example, if an intervention has a target recruitment of 50 participants, then recruiting all 50 would put them in green, recruiting less than this would put them in amber or red, see Fig. 1.

We began this process with a paper-based group exercise, asking participants to discuss each criteria in small teams (~ 5–6 people) with supporting materials. Figure 1 provides an example where a cut-off at 70% has been recommended, suggesting that interventions meeting < 70% of target (e.g. recruitment) would fall into red; described as ‘targets not being met’. Copies of this figure were provided to participants to support discussions.

Participants were then asked to use the electronic voting system to vote for the cut-off that they felt should be used to define when interventions move from amber to red for each of the progression criteria independently; (options were presented in increments of 5, between 40 and 100%).

### Post workshop questionnaire

**Fig. 2:**
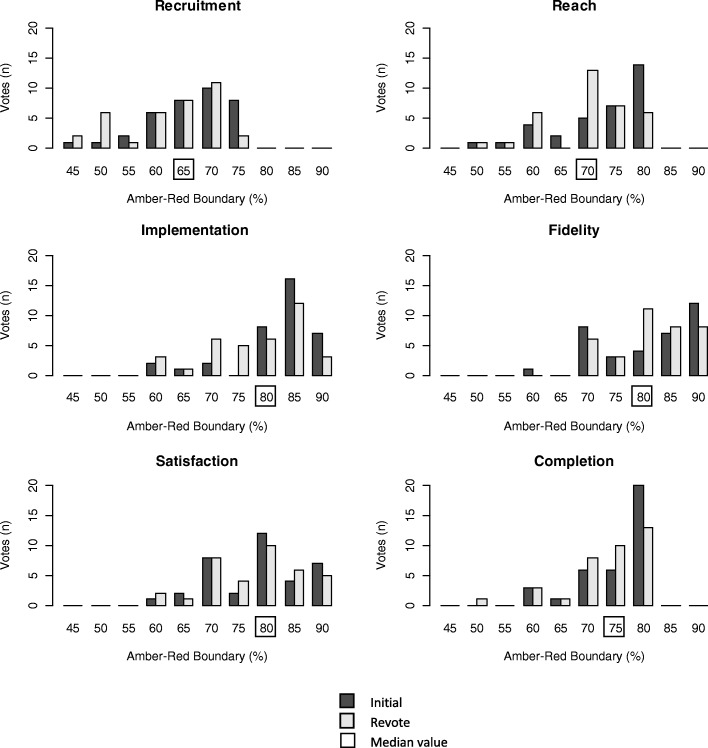
Frequency and the median value of votes for criteria cut-offs to indicate when interventions fall from amber (falling short of targets) to red (not meeting targets). For example, interventions which choose ‘recruitment’ as a key progression criteria will fall into ‘red’ if they recruit less than 65% of their target

Participants were given an opportunity to reflect upon the discussions and decisions made in the workshops and were invited to reconsider the amber/red boundary for each criterion in a post workshop questionnaire. A detailed summary document was circulated to all workshop participants, including details of what they had previously voted and the results of the workshop overall (i.e. frequency/proportion in which progression criteria were voted with the median and range of cut-offs for all workshop participants). Results from both the initial and the re-vote are provided in Fig 2, though a median of both was used in the final analysis.

### Analysis

To find the three key progression criteria for each intervention, electronic voting data from all participants were collated and the frequency with which each criterion ranked in each position was calculated per intervention. This was done by assigning a score to each rank position ranging from 1 to 6 (i.e. 6 points were allocated to a criterion if a participant considered it the most important, and 1 point was allocated if ranked as least important). This score was multiplied by the frequency of participants voting for each rank score to result in a total score for each criteria. Where three or more responses were missing for an individual participant within the ranking exercise for a single intervention, the complete vote was removed to ensure reliability of the calculated result. This process therefore takes account of all complete votes submitted, while weighting by rank position.

Data collected during the workshop to determine cut-offs were compared to responses to the post workshop questionnaire to determine whether participants felt differently upon reflection. We considered the frequency distribution for each criterion and calculated the median value. The median value was used to define cut-offs to incorporate the range of votes from the entire participant group.

Simple, univariate analyses were also conducted to explore voting patterns by stakeholder following both the ranking exercise and selection of cut-offs.

## Results

### Determining key progression criteria

Workshop participants unanimously agreed during discussions prior to formal voting that data quality should be a pre-requisite of all interventions. It was felt that this forms the foundation from which all other criteria can be judged. We therefore decided to exclude this as a potential progression criterion for the purposes of the ranking activities. Instead, the monitoring and review of all interventions require adequate data quality as standard progression criteria, and any reporting will feature a data quality summary to provide context.

Table 3 provides total scores from the ranking exercise to determine the top three progression criteria for each intervention. The criteria ranked highest across all interventions were recruitment and implementation, where one of these ranked in the top three criteria for every intervention, and both featured in the top three for more than half. Reach was also ranked high in interventions, placing in the top three criteria for six of the fourteen interventions.

**Table 3:**
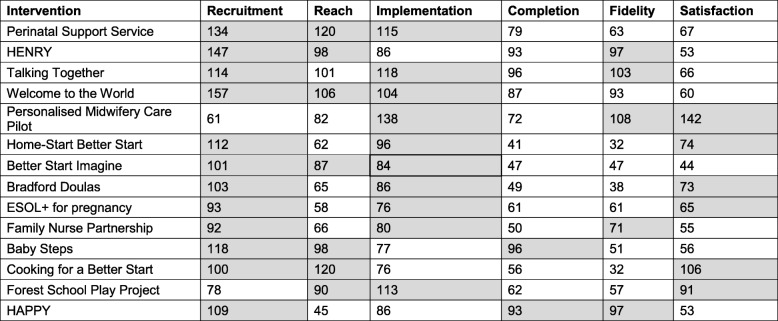
Progression criteria rank scores for all interventions

Scores assigned to each rank position were multiplied by the frequency of votes before being summed to result in a total score for each criteria; top three scores per project are highlighted

### Determining cut-offs to categorise performance of progression criteria

Voting for cut-offs was only performed in workshop one (*n* = 29). Fig. 2 shows the frequency and the median value of votes for cut-offs to select when each criteria would be considered as moving from Amber (falling short of targets) to Red (targets not being met, instigating actions to resolve issues and decommissioning discussions). As being Green (meeting targets) was always signified at 100%, participants only needed to consider the cut-off to indicate when an intervention moved from being Amber (less than 100%) to Red. Median cut-offs to indicate when interventions moved from Amber to Red ranged from 65% (recruitment) to 80% (implementation, satisfaction and fidelity). Thus for example, when recruitment falls less than 65% of its target, an intervention would be labelled as ‘not meeting targets’ (red); if recruiting between 65 and 99%, they would be considered as falling short of targets (amber); and 100% recruitment would indicate an intervention is meeting targets (green). We also introduced a further category of exceeding targets (shown in blue in Fig. 1), to highlight when interventions exceed their targets. The Fisher’s exact test revealed no significant difference in votes made between stakeholder groups for any criterion.

## Discussion

We applied a methodology recommended by the Medical Research Council to determine progression criteria to pilot and feasibility studies to support the monitoring of interventions being implemented in early years services [11]. Involvement of key stakeholders in the process led to joint decision making of and setting of, realistic targets where all parties are clear on the requirement of the service delivery and methods for monitoring. Further, this process ensures adequate and timely data collection and reduces ambiguity in the monitoring process. Perhaps expected, recruitment, implementation and reach were most highly ranked criteria in our workshops. However, rather than applying these criteria to all interventions, the teams were able to fully consider the key characteristics of each intervention and discuss what factors would be essential for it to be successfully delivered in order to improve outcomes.

To our knowledge, this is the first time that a systematic approach has been applied to the development and implementation of progression criteria to monitor public health interventions. However, it is somewhat analogous to the assessment of treatment fidelity, which is most common in trials of complex behavioural interventions. One exemplar of this work is the Fidelity Framework, developed by the National Institute of Health’s Behavioral Change Consortium, which includes the assessment and monitoring of public health interventions [14, 15]. Fundamental to this, is the scope to enhance treatment fidelity which is in line with our proposed progression criteria process. The Fidelity Framework provides a detailed checklist of the required attributes to assess the level of treatment fidelity in studies evaluating public health interventions at multiple stages from design to delivery. Within ‘Delivery’ of interventions, it advocates a priori specification of treatment fidelity (e.g. “providers adhere to delivering >80% of components”) and it is this aspect that our progression criteria methodology has focussed; ensuring clear criteria, with cut-offs that have been agreed upon by a range of stakeholders.

The key to successful implementation of progression criteria for any intervention is the approval of the definitions of criteria and the setting of measurable targets from which the progression can be based. For example, for the criteria of recruitment, the definition may be the number of families enrolled over a given period and the target could be 200 per year. Ideally, workshop and agreement of criteria should be done during the planning and organising service delivery stage (service design) prior to implementation, and reviewed on an annual basis. However, if interventions are already being delivered, commissioners should take time to discuss and agree targets for each of the chosen progression criteria. We involved the intervention teams and commissioners as participants in the workshops to determine progression criteria and provided them specific results to facilitate a discussion regarding how to define criteria and potentially reconsider of targets in their service level agreements. The concept of introducing criteria was generally well received.

Although our procedure was intended to provide evidence of which progression criteria were considered most important, the context behind decision making should also be considered. Thus, we recommend that final approval is given by commissioning teams in conjunction with the teams delivering the interventions. It remains true that decisions are made by personal, political and contextual factors and these need to be considered in conjunction with the decisions through workshops. Discussion with commissioners is also important to determine any nuances or discrepancies. For example, in one of the interventions (ESOL+ for pregnancy), the scores generated from the ranking exercise to determine the key criteria resulted in four, rather than three criteria (i.e. third and fourth ranked criteria were both scored equally). We took this information to the commissioning group to seek approval of which to use and it was decided that all four criteria would be monitored and reviewed after 12 months.

Given the resources required to run workshops (including a representative sample of stakeholders) to determine what criteria should be applied for progression, we examined the characteristics of the interventions reviewed in workshops one and two to determine whether an alternative, theory based method could be applied to future interventions to determine criteria. Our decision to do this was introduced after the first meeting and was not an a priori aim (hence not included in the methods). For this, we aimed to develop a decision tree model in which we identified common characteristics which led to the same decision making. For example, participants unanimously voted that recruitment should not be a key progression criteria for the statutory intervention (Personalised Midwifery Care Pilot). This was consistent in the workshop discussions and voting outcome. Thus, satisfaction, fidelity and implementation were voted as key criteria rather than recruitment. Similarly, in non-statutory, universally offered interventions, three common criteria were recruitment, implementation and reach. Whilst the majority of interventions were able to fit within the draft decision tree, the criteria of some interventions could not be predicted by the decision tree. We therefore decided to host the further third workshop in order to add precision in the design of a theory based decision tree to determine key progression criteria. However, even with data from the additional four interventions discussed in the third workshop, it was not possible to fit a decision making tree/model which would enable the progression criteria of other interventions without a workshop or discussion. Given the added value of involving stakeholders in the decision making, we therefore recommend that progression criteria for interventions not already determined here are chosen through a similar workshop process.

Given the potential consequences of decisions made following review of progression criteria, it is recommended that the frequency and methods for monitoring (which data are used, how frequently they are analysed, who should analyse them and how they are reported) should be agreed prior to data collection. In situations where multiple interventions are being monitored (such as with Better Start Bradford), interventions which consistently meet targets need less time dedicated to their monitoring and do not need to be escalated to commissioning leads. Instead, the progression criteria allows a greater discussion of interventions that are not meeting criteria to make improvements or consider de-commissioning in the context of other factors (e.g. it is possible that implementation targets are not being met due to staff illness or other issues outside of control of the intervention team).

**Fig. 3:**
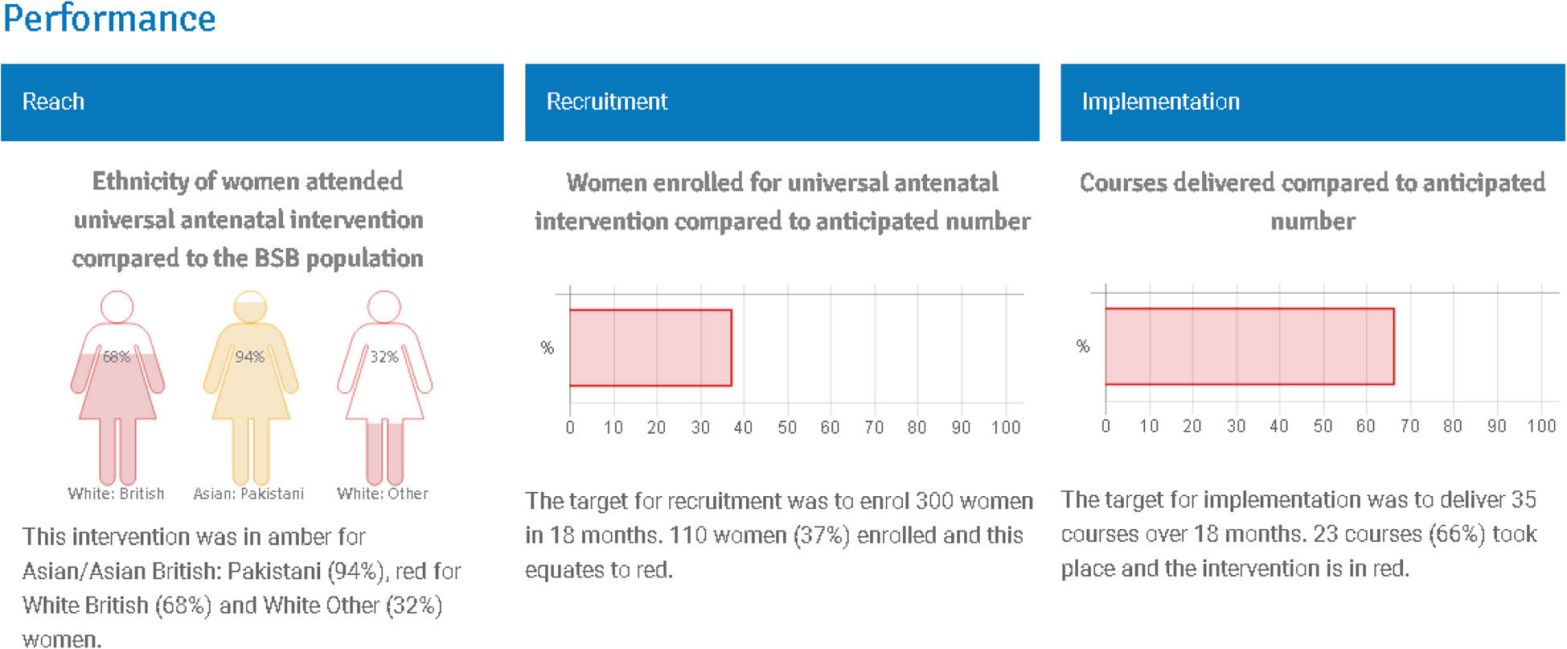
Presentation of progression criteria indicators

Figure 3 provides examples of how progression criteria are presented at monitoring meetings. This approach allows those who are monitoring interventions the ability to consider whether there are trends or inconsistencies in criteria over time. For example, if recruitment targets are not being met but appear to be improving over time, this can be considered in light of other processes that are being implemented to improve recruitment and may therefore be monitored rather than decommissioned. Similarly, if implementation targets are usually met, but then fall over a given period, contextual factors (e.g. staff sickness) should be considered. Further support on achieving successful implementation and monitoring of interventions can also be found here [16].

## Conclusion

Offering a method to select progression criteria means all commissioners can monitor more effectively, adapt early where needed and therefore more successfully implement much needed public health interventions; or decommission early ones which are not delivering. Though our work focused on early years interventions, it is anticipated that our methods can support the monitoring of other public health interventions to aid commissioning decision-making processes and provide support to services where needed.

## Data Availability

Data sharing is not applicable to this article.

## Abbreviations

BMI: Body Mass Index
BSB: Better Start Bradford
ESOL: English for Speakers of Other Languages
FNP: Family Nurse Parnership
HRA: Health Research Authority
MRC: Medical Research Council
